# An Iterative Module in the Azalomycin F Polyketide Synthase Contains a Switchable Enoylreductase Domain

**DOI:** 10.1002/anie.201701220

**Published:** 2017-04-18

**Authors:** Wei Xu, Guifa Zhai, Yuanzhen Liu, Yuan Li, Yanrong Shi, Kui Hong, Hui Hong, Peter F. Leadlay, Zixin Deng, Yuhui Sun

**Affiliations:** ^1^ Key Laboratory of Combinatorial Biosynthesis and Drug Discovery (Wuhan University) Ministry of Education, and School of Pharmaceutical Sciences Wuhan University 185 East Lake Road Wuhan 430071 P.R. China; ^2^ Department of Biochemistry University of Cambridge 80 Tennis Court Road Cambridge CB2 1GA UK

**Keywords:** antibiotics, biosynthesis, enoylreductases, iteration modules, macrocyclic polyketides

## Abstract

Detailed analysis of the modular Type I polyketide synthase (PKS) involved in the biosynthesis of the marginolactone azalomycin F in mangrove *Streptomyces* sp. 211726 has shown that only nineteen extension modules are required to accomplish twenty cycles of polyketide chain elongation. Analysis of the products of a PKS mutant specifically inactivated in the dehydratase domain of extension‐module 1 showed that this module catalyzes two successive elongations with different outcomes. Strikingly, the enoylreductase domain of this module can apparently be “toggled” off and on : it functions in only the second of these two cycles. This novel mechanism expands our understanding of PKS assembly‐line catalysis and may explain examples of apparent non‐colinearity in other modular PKS systems.

Modular Type I polyketide synthases (PKSs) catalyze the biosynthesis of numerous pharmacologically relevant natural products that exhibit antibacterial, antifungal, anthelmintic, antitumor, or immunosuppressive activities.^1^, ^2^, ^3^, ^4^ These giant multimodular enzymes form a processive assembly‐line to produce the polyketide backbone using (alkyl)malonyl‐CoA esters as the source of extender units. Each module consists of a β‐ketoacyl synthase (KS) domain for condensing the incoming unit onto the growing polyketide chain, an acyltransferase (AT) domain for loading extension units, and an acyl carrier protein (ACP) domain for retention of the growing polyketide chain on the PKS. In addition to these conserved domains, β‐ketoreductase (KR), dehydratase (DH), and enoylreductase (ER) domains are optional to achieve varying degrees of reduction. Finally, the full‐length chain is released, for example by a cyclase/thioesterase (TE).^5^ In general, the organization of modules and domains corresponds exactly to the chemical structure of the initial polyketide product. This colinear property has made modular PKSs attractive subjects for rational bioengineering to produce novel bioactive compounds,^6^ and has facilitated the “mining” of genome sequences for novel biosynthetic pathways.^7^, ^8^, ^9^ However, there is growing interest in exceptions to this rule. An early example is the pikromycin PKS, which produces both a 12‐membered and a 14‐membered macrolide through optional “skipping”^10^ of a PKS module. In other examples, a single extension module catalyzes two or more successive (and identical) rounds of polyketide carbon skeleton, either to give aberrant products^11^ or as part of the normal biosynthetic pathway.^12^, ^13^, ^14^, ^15^, ^16^ These exceptions promise to increase our understanding of the natural evolution of modular PKSs, and potentially offer lessons for the effective engineering of these systems.

The 36‐membered macrocyclic antifungal azalomycins (AZLs) are the main products of *Streptomyces* sp. 211726, which has been isolated from mangrove rhizosphere soil.^17^, ^18^ These compounds show broad‐spectrum antimicrobial activity, and cytotoxicity against a human colon tumor cell line.^17^, ^18^ Azalomycin F5a and certain F5a derivatives also show anti‐MRSA activity.^19^ Azalomycins F3a, F4a, and F5a are also produced by *Streptomyces malaysiensis* DSM 4137,^20^ and we have previously proposed a model in which the first extension module catalyzes both the first and second cycles of polyketide chain extension, based on the observation that when the cluster is transplanted into a heterologous strain, azalomycins are produced, which rules out the participation of an additional PKS encoded elsewhere in the *S. malaysiensis* genome.^21^ Whole‐genome sequencing of *Streptomyces* sp. 211726 revealed a cluster (GenBank accession number: KY484834) that is highly similar, both in size and organization, to the azalomycin cluster in DSM 4137.^20^ It has exactly the same module and domain arrangement and flanking auxiliary genes spanning an approximately 130 kb region of DNA (Figure 1 and Table S1 in the Supporting Information), and deletion of the entire region (Figure S1 in the Supporting Information) led to total loss of AZL production. As before, the loss of colinearity is readily localized to modules 1 and 2 (Figure 2 and Figure S2).

**Figure 1:**
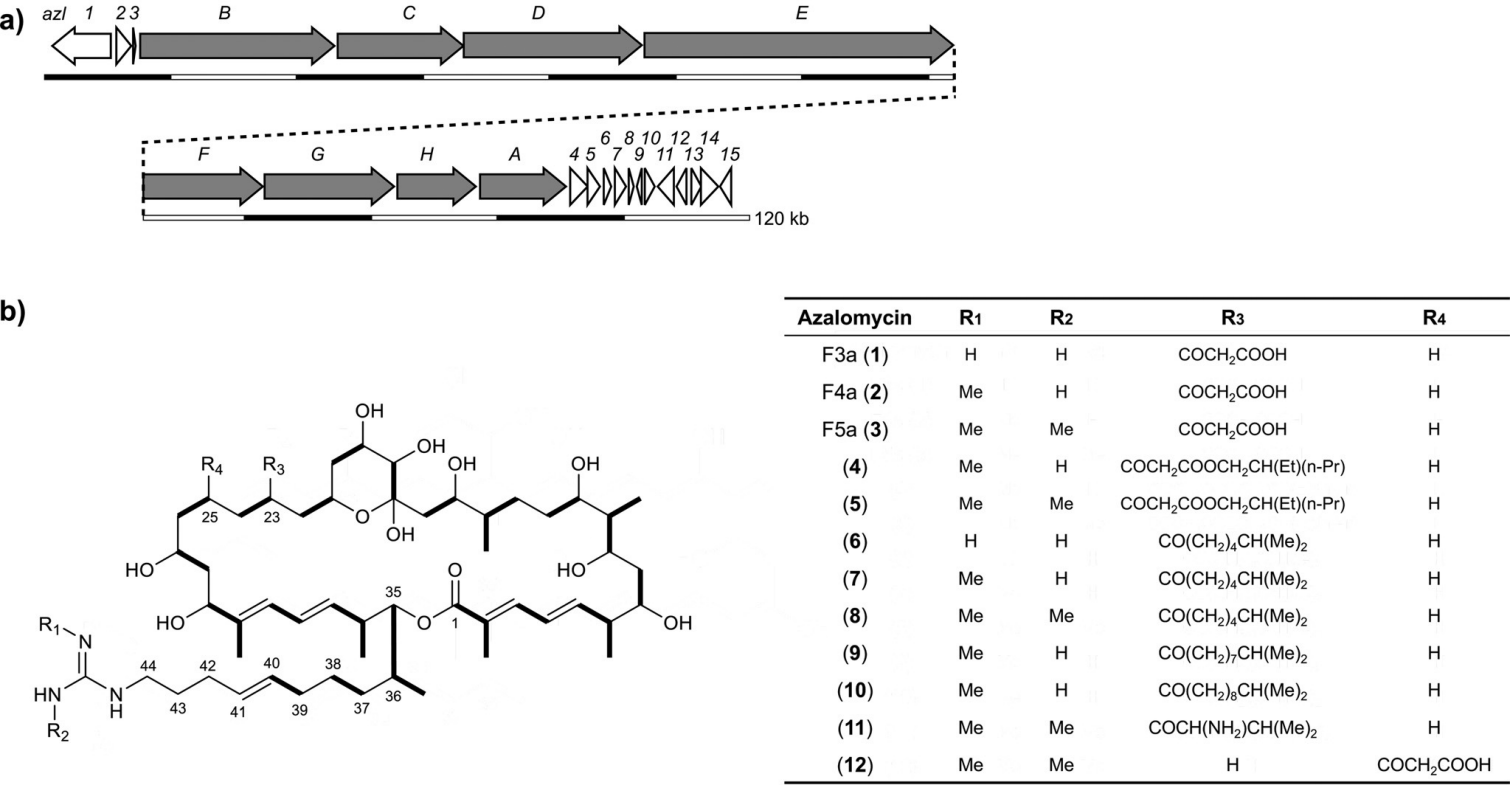
a) Organization of the azalomycin F biosynthetic gene cluster. The PKS‐encoding genes are highlighted in gray. b) Structures of azalomycin F analogues. Bold lines indicate the malonyl and methylmalonyl extender units incorporated by each acyltransferase domain of the PKS. Compounds **4**–**12** are minor components.

**Figure 2:**
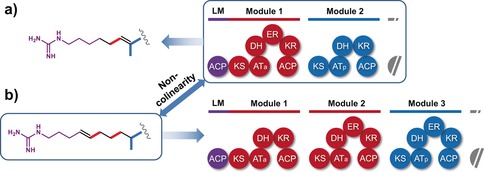
Non‐colinearity between the AZL PKS assembly line and the product structure. a) Proposed chemical structure of AZL (the guanidino‐substituted chain is shown) deduced from the nucleotide sequence and the predicted domain organization of the AZL biosynthetic gene cluster. b) Bioinformatic prediction of PKS organization based on the actual structure of AZL according to the canonical colinearity rule.

In order to test the hypothesis that extension‐module 1 acts iteratively and supplies the missing full reduction module governing the second extension, a site‐specific mutation of the essential histidine residue to alanine was introduced into the active site of the DH_1_ domain in vivo (Figure S3). Liquid chromatography electrospray ionization high‐resolution mass spectrometry (LC‐ESI‐HRMS) analysis showed that the ΔDH_1_ mutant strain no longer produces known AZLs, and instead significant new peaks were found (Figure 3 c and Figure S4), albeit at levels only 0.1 % of AZL levels in the wild‐type. Analysis of these new peaks revealed compounds closely related to azalomycins F3a, F4a, and F5a, except that they bear hydroxy groups at C‐41 and C‐39, instead of a double bond between C‐40 and C‐41 (Figure 3 c and Figure S4). These compounds, named F3a′, F4a′, and F5a′, were isolated from a large‐scale fermentation, and their structures were elucidated by ^1^H NMR and DEPT spectroscopy. The NMR data showed in each case the loss of two olefinic protons (δ_H_ 5.38–5.46, 2 H) and the presence of two additional O‐bound protons at δ_H_ 3.65 and δ_H_ 4.04 (Figure S5) compared to the known azalomycins. In agreement with this, the DEPT analysis showed a lack of two olefinic carbon atoms at δ_C_ 128.9 and δ_C_ 131.0 Figure S6). Clearly, inactivation of the DH_1_ domain prevents dehydration in both the first and second extensions, thus providing direct evidence that in the AZL PKS, module 1 is used twice.

**Figure 3:**
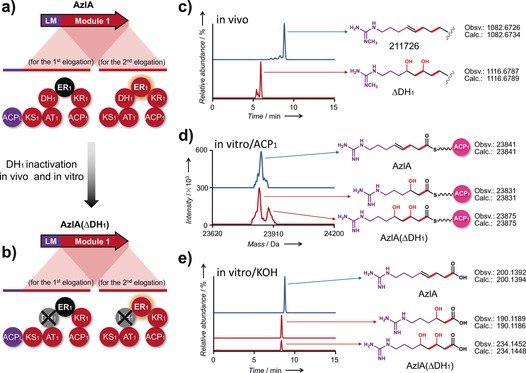
A model for the toggling ER_1_ domain in iterative‐module 1 based on the results of inactivation of the DH_1_ domain in vivo and in vitro. a) Domain organization of AzlA. Module 1 is used twice, and the toggling ER_1_ domain is programmed to be inactive (shaded in black) in the first extension but active in the second extension. b) Domain organization of AzlA(ΔDH_1_). The DH_1_ domain marked with a cross was inactivated by site‐specific mutagenesis in the 211726 chromosome and the AzlA recombinant multienzyme. c) LC‐ESI‐HRMS analysis of fermentation products from wild‐type 211726 and the ΔDH_1_ mutant. Only F4a and its derivative F4b, the main products of AZL, are shown. d) LC‐ESI‐HRMS protein analysis of an in vitro reconstitution assay of AzlA and AzlA(ΔDH_1_) with added recombinant stand‐alone ACP_1_. e) LC‐ESI‐HRMS analysis of the same in vitro assay mixture as in (d) except that the reaction mixture was subjected to alkaline hydrolysis before analysis. The three traces are presented on the same scale.

The PKS AzlA houses only the loading module (LM) and extension‐module 1. To further confirm the above result by in vitro analysis, the recombinant PKS AzlA(ΔDH_1_), which bears the same site‐directed mutation in the DH_1_ domain as above, was successfully expressed in soluble form in *Escherichia coli* BAP1^22^ with post‐translational modification of the ACP_1_ domain. In a one‐pot reaction with 4‐guanidinobutyric acid, purified Azl4 (ligase), Azl5 (acyltransferase), and AzlA(ΔDH_1_), the starter unit 4‐guanidinobutyryl‐CoA was produced and loaded onto the ACP_L_ domain that comprises the LM in AzlA(ΔDH_1_). In order to achieve multiple turnovers of polyketide synthesis on the AzlA PKS multienzyme, our strategy^23^ was to supply additional copies of the ACP_1_ domain. Recombinant *holo*‐ACP_1_ domain was expressed as a stand‐alone protein and purified from *E. coli* BAP1 and added to the incubations to compete with the integral ACP_1_ domain of module 1 (Figure S7). The polyketide‐chain‐bearing ACP_1_ reaction products were monitored by LC‐ESI‐HRMS. In the presence of *holo*‐ACP_1_ and malonyl‐CoA in the above one‐pot reaction, a peak with a mass corresponding to the expected product of two rounds of extension (8‐guanidino‐3,5‐dihydroxyoctanoyl‐ACP_1_) was observed (Figure 3 d). A further peak was detected corresponding to the mass of 6‐guanidino‐3‐hydroxyhexanoyl‐ACP_1_, the expected product of a single cycle of chain extension (Figure 3 d). To confirm these findings, thioester‐bound products were hydrolyzed with potassium hydroxide as described^24^ in both assays with and without *holo*‐ACP_1_ and showed the same result. LC‐ESI‐HRMS analysis of the hydrolyzed products revealed two peaks, corresponding to 6‐guanidino‐3‐hydroxyhexanoic acid and 8‐guanidino‐3,5‐dihydroxyoctanoic acid, respectively, which is in agreement with the analysis of the acyl‐ACP_1_ species (Figure 3 e). Taken together, these results demonstrate conclusively that the AZL PKS extension‐module 1 catalyzes two successive cycles of chain extension. In the iterative mechanism we propose for the intact AZL PKS (Figure S7), after the first round of extension, the ACP_1_‐tethered polyketide intermediate is transferred back to the KS_1_ domain on the opposite PKS strand^25^ instead of the downstream KS_2_ domain, probably owing to the preference of KS_2_ for the triketide acyl‐ACP as a substrate over the diketide acyl‐ACP.

Notably, only one full reduction occurs during the first two extension cycles on the AZL PKS. To investigate the behavior of the ER_1_ domain in module 1, the same experimental approach as above, in which recombinant ACP_1_ was used in a one‐pot reaction with wild‐type AzlA (Figure S7), was used. Since module 1 contains a full set of reductive enzyme domains (DH, ER, KR), and recruits malonyl‐CoA as extender unit, the 4‐guanidinobutyryl starter unit should be extended with two acetate units and both newly‐formed β‐keto groups should be fully reduced. However, LC‐ESI‐HRMS analysis of the one‐pot reaction showed that the molecular weight of the acyl‐ACP_1_ species produced was 2 Da less than expected for the product of two rounds with full reduction (Figure 3 d), thus suggesting incomplete reduction in one cycle. To confirm this, analysis of the acyl chain after its release from ACP_1_ by alkaline hydrolysis showed it to be 8‐guanidinooct‐4‐enoic acid (Figure 3 e). Therefore, AzlA catalyzes two elongations, in only one of which the enoylreductase (ER) domain acts. The position of the double bond in all natural AZL compounds (between C‐40 and C‐41) is fully consistent with ER_1_ being inactive in the first extension but active in the second one.

The programmed iteration of extension‐module 1 on the AZL PKS occurs, as with previously identified examples of programmed iteration of modules,^11^, ^26^ at a point in the assembly line where there is a protein–protein interface^27^ (here between AzlA and AzlB). Those previous examples all involve the same level of β‐keto processing in each of the iterative rounds of chain elongation. Here, we report an unprecedented example in which every domain except the ER is used during two successive rounds of elongation, while the ER domain is non‐functional (“off”) in the first but functional (“on”) in the second extension process. Nevertheless, the iterative use of module 1 and the ER_1_ skipping event in the first extension can both be accounted for in terms of kinetic control: the intrinsic selectivity of the ER_1_ domain, which discriminates against the shorter substrate, would favor back‐transfer of the acyl chain onto KS_1_. Likewise, for the KS_1_ active site to be re‐used, the back‐transfer must outcompete the priming of KS_1_ through transfer of the 4‐guanidinobutyryl starter unit from the adjacent ACP_L_ loading domain. After two rounds of extension, the partitioning of the triketide acyl‐ACP intermediate favors full reduction rather than transfer to the next module. The downstream KS_2_ domain on the adjacent PKS subunit AzlB may well act as a “gatekeeper”, favoring selective recruitment of a triketide‐ rather than a diketide‐acyl chain. Evidence from a study of the aureothin PKS^28^ strongly supports such an interplay of multiple factors in programmed iteration.

Selective processing during two successive iterations of polyketide chain extension is a known feature in highly reducing fully iterative polyketide synthases.^29^, ^30^, ^31^ In the LovB PKS, for example, which synthesizes the nonaketide core of the cholesterol‐lowering compound lovastatin using a single PKS module, an integral methyltransferase domain selectively methylates the β‐ketothioester formed after three rounds of chain extension.^29^ In vitro analysis of model thioester substrates for LovB has demonstrated that the methyltransferase is exquisitely specific for the tetraketide substrate, and that for this substrate, methylation effectively outcompetes prior ketoreduction.^32^ LovB also recruits an exogenous monomeric ER protein (LovC) to catalyze specific enoyl reduction in only three out of the eight extension cycles (tetra‐, penta‐, and heptaketide intermediates).^32^ Detailed analysis of LovC has suggested that while the competent substrates are readily accommodated in a productive conformation, shorter intermediates preferentially adopt non‐productive conformations in the active site.^32^ Meanwhile, the hexaketide intermediate preferentially undergoes Diels–Alder‐like cyclization. Similar experiments will be needed to establish the precise structural basis for the remarkable switch that flips the AzlA ER_1_ domain from “off” to “on” during azalomycin biosynthesis.

## Conflict of interest

The authors declare no conflict of interest.

## Supporting information

As a service to our authors and readers, this journal provides supporting information supplied by the authors. Such materials are peer reviewed and may be re‐organized for online delivery, but are not copy‐edited or typeset. Technical support issues arising from supporting information (other than missing files) should be addressed to the authors.

Supplementary
