# Supplementary material for: An Iterative Module in the Azalomycin F Polyketide Synthase Contains a Switchable Enoylreductase Domain
Source: Angew Chem Int Ed Engl. 2017 Apr 18;56(20):5503–6. doi: 10.1002/anie.201701220 (PMC5518293; doi:10.1002/anie.201701220)
Supplement: Supplementary file 1 — Supplementary [file ANIE-56-5503-s001.pdf]

Angewandte Chemie
Eine Zeitschrift der Gesellschaft Deutscher Chemiker

Supporting Information

An Iterative Module in the Azalomycin F Polyketide Synthase Contains a Switchable Enoylreductase Domain

*Wei Xu[+], Guifa Zhai[+], Yuanzhen Liu, Yuan Li, Yanrong Shi, Kui Hong, Hui Hong, Peter F. Leadlay, Zixin Deng, and Yuhui Sun**

anie_201701220_sm_miscellaneous_information.pdf

Bacterial strains, plasmids and DNA manipulation. Bacterial strains and plasmids used during this study are summarized in Supporting Information, Table S2. DNA manipulations were performed using standard procedures for *E. coli* and *Streptomyces*. All chemical reagents and antibiotics were purchased from Sigma-Aldrich. All oligonucleotide primers used in this study (Supporting Information, Table S3) were synthesized by Tsingke. DNA sequencing of PCR products was performed by Tsingke.

Culture conditions. *Streptomyces* sp. 211726 wild-type and mutants were grown in TSBY liquid medium (3% tryptone soy broth, 10.3% sucrose, 0.5% yeast extract) for isolating chromosomal DNA, and SFM medium (2% mannitol, 2% soya flour, 2% agar) for AZL production. For liquid culture, the strains were grown at 28°C and 220 rpm on a rotary incubator and harvested after 3-4 days, and for solid culture, the strains were grown at 28 °C for 7-10 days. *E. coli* strains were cultured in 2×TY (1.6% tryptone, 1% yeast extract, 0.5% NaCl) medium at 37 °C with the appropriate antibiotic selection at a final concentration of 100 μg ml^{-1} carbenicillin, 50 μg ml^{-1} apramycin, 50 μg ml^{-1} kanamycin, 25 μg ml^{-1} chloramphenicol, or 25 μg ml^{-1} nalidixic acid as appropriate.

Generation of gene library. To obtain cosmids covering the *azl* gene cluster, a cosmid library was constructed. *Streptomyces* sp. 211726 total DNA was highly random sheared into about 40 kb by pipette, end-repaired and size-selected by pulsed-field gel electrophoresis. The appropriate size DNA was recovered and ligated to pre-linearized pCC1FOS vector (Epicentre, # CCFOS110) and packaged with MaxPlax Lambda Packaging Extracts

(Epicentre, # MP5105). All procedures were carried out in accordance with the manufacturer's recommendations.

Procedure for gene disruption in vivo. The constructs used for gene disruption were introduced into *Streptomyces* sp. 211726 by conjugation using donor strain ET12567/pUZ8002 on SFM plates. After incubation at 28°C for 16 h, exconjugants were selected on SFM plates with 25 μg ml^{-1} apramycin and 25 μg ml^{-1} nalidixic acid. Single exconjugants were transferred to an SFM plate containing 50 μg ml^{-1} apramycin and 25 μg ml^{-1} nalidixic acid to confirm their antibiotic resistance. They were then patched onto SFM plates containing 50 μg ml^{-1} apramycin and onto SFM plates without antibiotic, respectively, to screen for the double crossover mutant. Candidate mutants with the correct phenotype (Apr^S) were further verified by PCR, sequencing or Southern blot.

Production, isolation and LC-ESI-HRMS analysis of AZL. To obtain AZL production by *Streptomyces* sp. 211726 and mutants, 40 ml TSBY liquid medium was inoculated and grown as seed culture. After 2-3 days, 7 ml seed mycelium was inoculated into 700 ml SFM liquid medium and grown as described above. After 10 days, the supernatant was extracted with 700 ml ethyl acetate, and the mycelium was extracted with 500 ml methanol. The combined organic phase from both the culture supernatant and the mycelium (using both ethyl acetate and methanol extraction) was evaporated to dryness under reduced pressure using a Speed-Vac, then subjected to chromatography on Sephadex LH-20 (40-70 μm) and on a Phenomenex Synergi C18 column (250×10 mm, 4 μm) for separation and purification of AZL and derivatives.

Purified AZL and derivatives were analyzed by liquid chromatography-electrospray ionization-high resolution mass spectrometry (LC-ESI-HRMS). On-line LC-ESI-HRMS analysis was carried out on a Thermo Electron LTQ-Orbitrap XL using positive-mode electrospray ionization. The LTQ-Orbitrap XL was coupled to Thermo Accela 600 fitted with a Phenomenex Luna C18 column (250×4.6 mm, 5 μm) at a flow rate at 1 ml min^{-1}. The gradient for separation of AZL from crude sample: 0 min 90% 0.1% formic acid in H_2O (A) and 10% 0.1% formic acid in acetonitrile (B), 0-2 min 10% B to 80% B, 2-10 min 80% B to 95% B, 10-11 min 95% B, 11-12 min 95% B to 10% B, 12-15 min 10% B. The mass spectrometer was set to full scan (from 200 to 2000 *m/z*). To obtain enough of the AZL and derivatives to be characterized by NMR spectra, wild-type 211726 and ΔDH mutant were fermented on a larger scale. 1.5 l seed culture was inoculated to 40 l SFM liquid medium. After 5-day fermentation at 28˚C, same procedure was performed to obtain desired compounds. About 15 mg compound was obtained from 40 l fermentation.

Preparation of gene disruption constructs. To disrupt the *azl* gene cluster, two homologous recombination fragments 2069 bp and 2001 bp flanking the ~120 kb *azl* cluster in the genome were amplified by PCR using primer pairs Δazl-L1 and Δazl-L2, Δazl-R1 and Δazl-R2, respectively. After digestion with appropriate restriction enzymes, the two fragments were cloned into *Streptomyces-E. coli* shuttle vector pYH7[1] treated with *Nde*I and *Hin*dIII by three piece ligation to create pWHU2790. To verify the in-frame deletion in the construct and the mutant, a pair of primers Δazl-CP1 and Δazl-CP2 flanking the target gene were used for PCR and sequencing.

Site-directed mutation of the DH_1 domain. To inactivate the DH_1 domain by site-directed mutagenesis in vivo, a 11592 bp *Kpn*I-digested fragment harboring the *azlA* gene from cosmid 3G11 was ligated to *Kpn*I and FastAP-treated pYH7[1] vector. Two fragments were amplified from the above recombinant plasmid using primer pairs ΔDH-L1 and ΔDH-L2, ΔDH-R1 and ΔDH-R2, and fused into one fragment by overlapping PCR, to obtain the site-directed mutation donor. The recombinant plasmid was mutated by the donor via PCR-targeting[2] in BW25113/pIJ790 to generate pWHU2791, the final construct for site-directed mutation of DH_1 domain. To verify the site-directed mutation in construct and mutant, a pair of primers ΔDH-CP1 and ΔDH-CP2 flanking the target site were used for PCR and sequencing.

Characterization of AZL derivatives by NMR and LC-ESI-HRMS. The ^{1}H-, and DEPT NMR spectra were collected on an Agilent 400MR DD2 NMR spectrometer. Chemical shifts were reported in ppm using tetramethylsilane as an internal standard, and NMR data processing was performed by using MestReNova software. LC-ESI-HRMS analysis was performed on a Thermo Electron LTQ-Orbitrap XL mass spectrometer using the method described above.

Preparation of protein expression constructs. To generate the Azl4 expression construct, amplification of *azl4* was accomplished with primers exAzl4-F and exAzl4-R. The PCR product was cloned into pET28a(+) to yield pWHU106. Similarly, the constructs pWHU107, pWHU86, pWHU2796 were

generated by using primer pairs exAzl5-F and exAzl5-R for expression of Azl5, exAzlA-F and exAzlA-R for expression of AzlA, exACP1-F and exACP1-R for expression of ACP_1 domain, respectively. To generate the AzlA(ΔDH_1) expression construct, amplification was accomplished by using the same expression primers of AzlA. The PCR product was cloned into pET28a(+) to yield pWHU2793.

Expression and purification of proteins. To express Azl4 and Azl5, appropriate plasmids were transformed into *E. coli* strain BL21(DE3), respectively, and grown in 2×TY medium supplemented with kanamycin (50 μg ml^{-1}). For expression of *holo*-AzlA, *holo*-AzlA(ΔDH_1) and *holo*-ACP_1, the host strain was *E. coli* BAP1. Each liter of culture was inoculated with 10 ml overnight starter culture and grown at 37 °C to A_{600} 0.3-0.5. Isopropyl-β-D-thiogalactopyranoside (IPTG) was then added at 0.1 mM, and incubation was continued at 16 °C for an additional 18 h. Cells were harvested by centrifugation (5000×*g* for 10 min), suspended in lysis buffer (20 mM Tris-HCl buffer pH 7.9, containing 50 mM NaCl and 5 mM imidazole), and lysed by sonication. The lysate was clarified by centrifugation (17000×*g* for 30 min). His-Bind resin (2 ml slurry per 1 l of culture) was added to the clarified lysate and allowed to bind at 4°C for 30 min. The resin was washed with five column volumes of lysis buffer. Bound proteins were then eluted with a step gradient of increasing imidazole concentrations (50, 100, 200, 400 and 800 mM in lysis buffer). Fractions containing purified protein were pooled and concentrated based on SDS-PAGE and LC-ESI-HRMS analysis (Supporting Information, Figure S8). The

theoretical molecular weights of Azl4, Azl5, *holo*-AzlA, *holo*-AzlA(ΔDH_1) and *holo*-ACP_1 are 54023, 40856, 244455, 244389 and 23660 Da, respectively.

Azl4 assay. The reaction was performed in a total volume of 500 μl containing 10 μM Azl4, 10 mM 4-guanidinobutyric acid, 1 mM CoA, 5 mM ATP and 5 mM $MgCl_2$ in SH buffer (50 mM HEPES, 100 mM NaCl, pH 7.5) at 30 ˚C for 30 min. The reaction was stopped by denaturation of proteins with chloroform and the aqueous phase was directly analyzed by LC-ESI-HRMS.

AzlA assay. To assay for polyketide chain elongation, reaction was performed in a total volume of 500 μl containing 2 μM Azl4, 2 μM Azl5, 2 μM *holo*-AzlA, 10 μM *holo*-ACP_1 1 mM 4-guanidinobutyric acid, 600 μM malonyl-CoA, 1 mM CoA, 5 mM ATP, 2 mM NADPH, 5 mM TCEP and 10 mM $MgCl_2$ in potassium phosphate buffer (pH 7.4) at 30˚C overnight. After reaction, the solution was directly analyzed by LC-ESI-HRMS. In reaction mixtures without added *holo*-ACP_1, the release of polyketide chains in thioester linkage to AzlA was accomplished by hydrolysis as described below, and the products were submitted to LC-ESI-HRMS analysis.

LC-ESI-HRMS analysis of assay. Analysis of enzymatic reaction products was carried out on a Thermo Electron LTQ-Orbitrap XL using positive-mode electrospray ionization. The LTQ-Orbitrap XL was coupled to Thermo Accela 600 fitted with a Thermo Hypersil GOLD C4 column (100×2.1 mm, 1.9 μm) at a flow rate at 0.1 ml min^{-1}. The gradient for separation of protein: 0-1 min 95% 0.1% trifluoroacetic acid (TFA) in H_2O (A) and 5% 0.1% TFA in acetonitrile (B),

1-20 min 5% B to 95% B, 20-25 min 95% B, 25-27 min 95% B to 5% B, 27-30 min 5% B. The mass spectrometer was set to full scan (from 300 to 2000 *m/z*). The mass spectrometric data were processed and deconvoluted using the Bioworks software (Thermo Finnigan).

Hydrolysis of thioester-bound products and LC-ESI-HRMS analysis. After enzymatic assay, the reaction was quenched by the addition of 10% trichloroacetic acid (TCA), and precipitated protein was pelleted by centrifugation and was washed twice with 10% TCA. The protein pellet was then dissolved in KOH (0.1 M, 100 μl) and heated at 65°C for 5 min. Trifluoroacetic acid (TFA) (50%, 5 μl) was then added, and the solution was centrifuged to remove precipitated proteins. The solution was applied to LC-ESI-HRMS for analysis. On-line LC-ESI-HRMS analysis was carried out on a Thermo Electron LTQ-Orbitrap XL using positive-mode electrospray ionization. The LTQ-Orbitrap XL was coupled to Thermo Accela 600 fitted with a Phenomenex Luna C18 column (250×4.6 mm, 5 μm) at a flow rate at 0.4 ml min^{-1}. The gradient for separation of alkaline hydrolysis products is: 0 min 90% 0.1% formic acid in H_2O (A) and 10% 0.1% formic acid in acetonitrile (B), 0-2 min 10% B to 80% B, 2-10 min 80% B to 95%, 10-11 min 95% B, 11-12 min 95% B to 10%, 12-15 min 10% B. The mass spectrometer was set to full scan (from 100 to 1000 *m/z*).

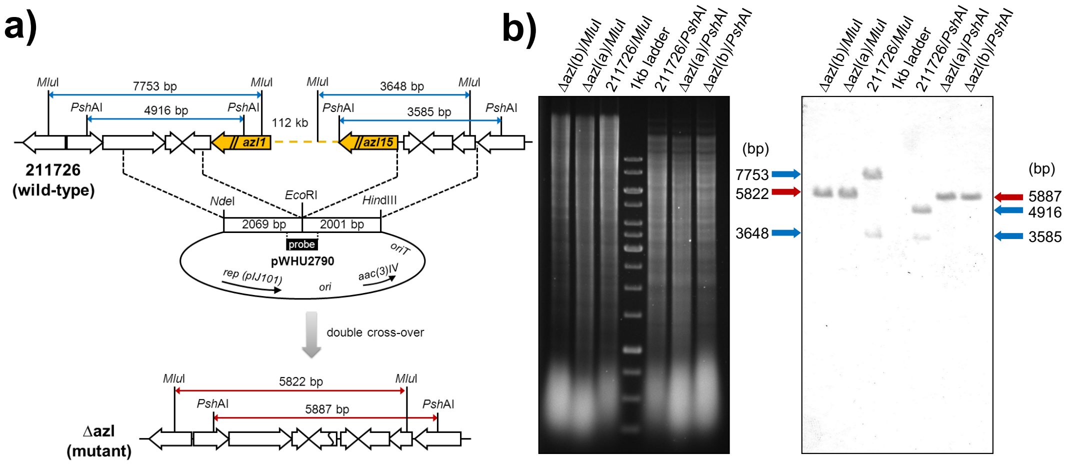

Figure S1. Schematic representation and confirmation of *azl* gene cluster deletion by Southern blot. The blue and red double-headed/solid arrows indicate the expected size of the *Mlu*I or *Psh*AI digested fragments from the wild-type and mutant chromosomal DNA, respectively, when hybridized with a 509 bp probe PCR-amplified from pWHU2790 by primers Δazl-CP1 and Δazl-CP2.

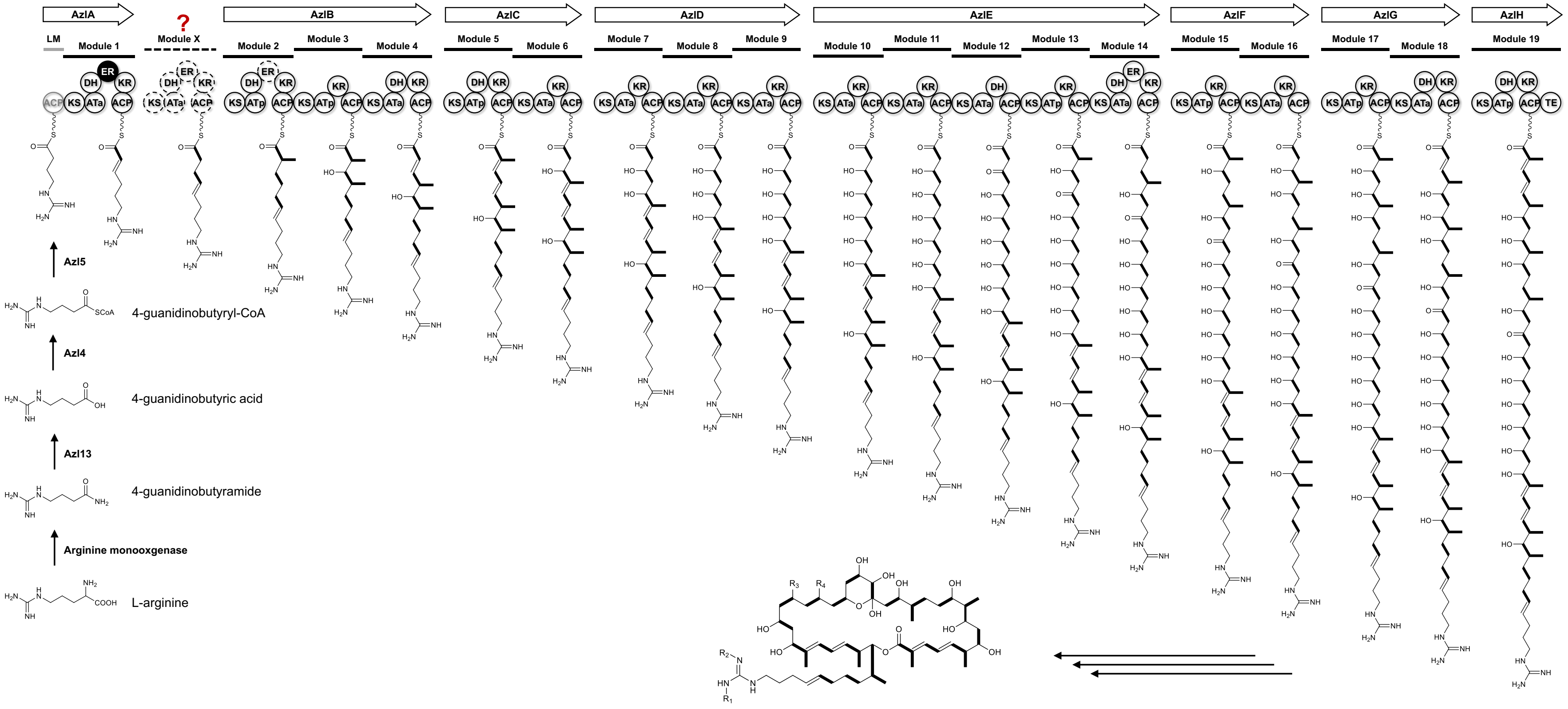

Figure S2. Putative diagram for AZL biosynthesis. Each circle represents an enzymatic domain in the PKS, in which particularly ATa indicates the domain loading a malonyl-CoA to build acetate unit (bold single line) and ATp to load a methylmalonyl-CoA to build propionate unit (bold fold line), respectively. Notably, the dotted circles/line indicate the missing domains/module which should be present according to the known AZL structure.

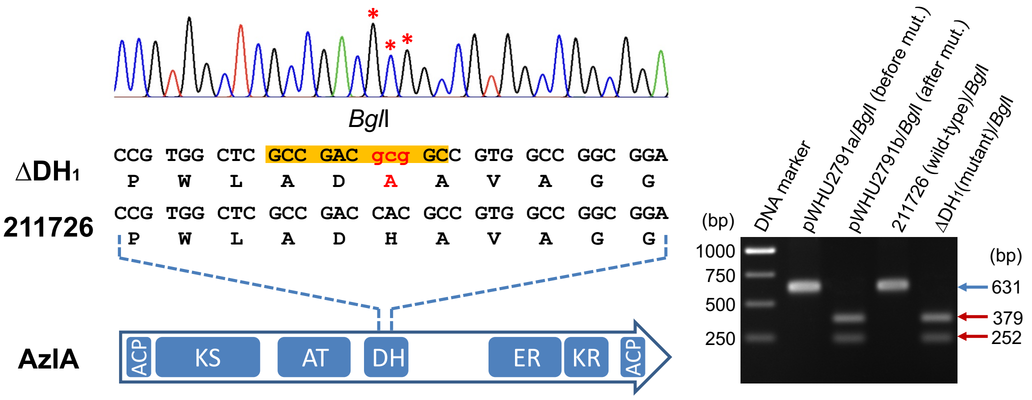

Figure S3. Inactivation of the DH_1 domain by site-directed mutation and confirmation. The histidine residue of DH_1 active site was mutated to alanine, shown in red or marked with asterisks, resulting in the appearance of a *Bgl*I restriction site which is highlighted with yellow. The mutation was confirmed by restriction enzyme digestion and sequencing.

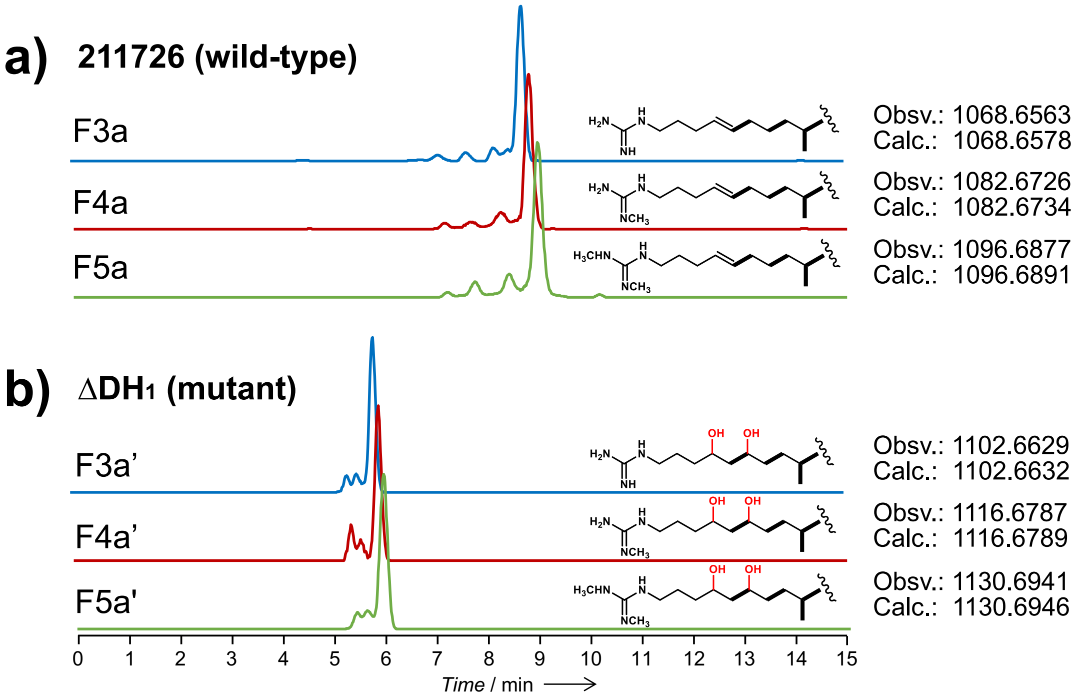

Figure S4. LC-ESI-HRMS analysis of fermentation products from ΔDH_1 mutants. The main azalomycin products of a) *Streptomyces* sp. 211726, F3a, F4a and F5a, and b) corresponding derivatives F3a', F4a' and F5a' from ΔDH_1 mutants were presented as extracted ion chromatogram.

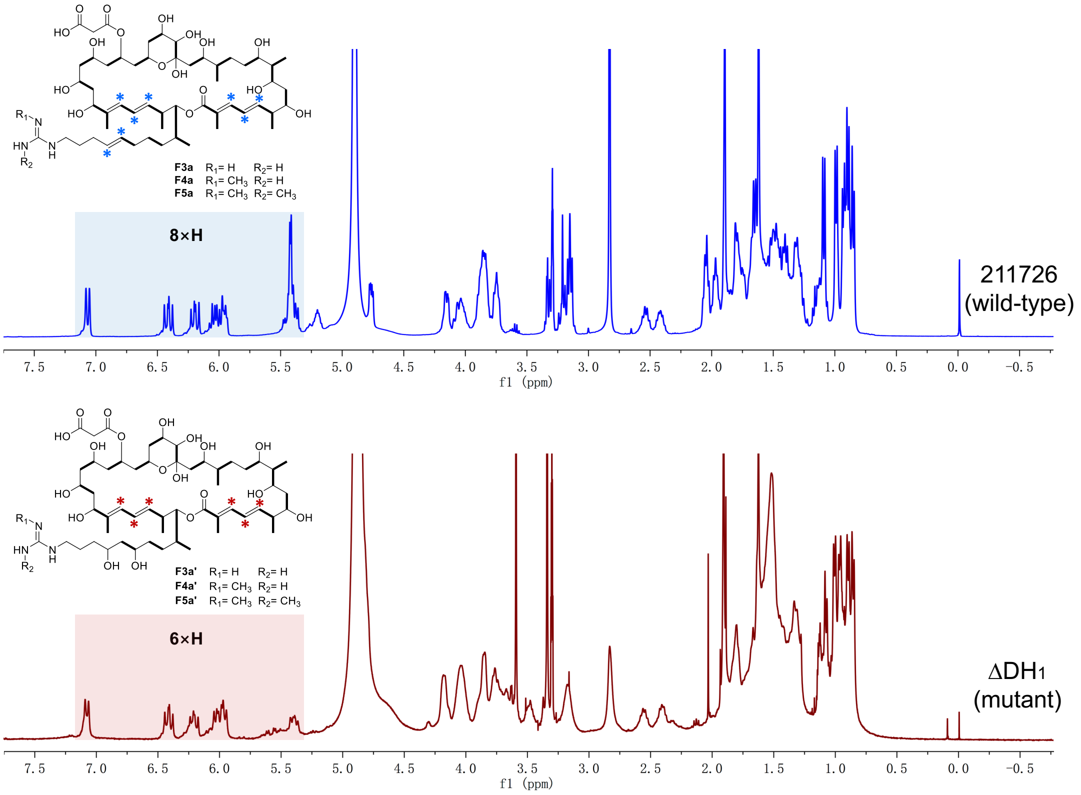

Figure S5. Comparison of ^{1}H NMR spectra between AZL and derivatives produced by ΔDH_1 mutant.

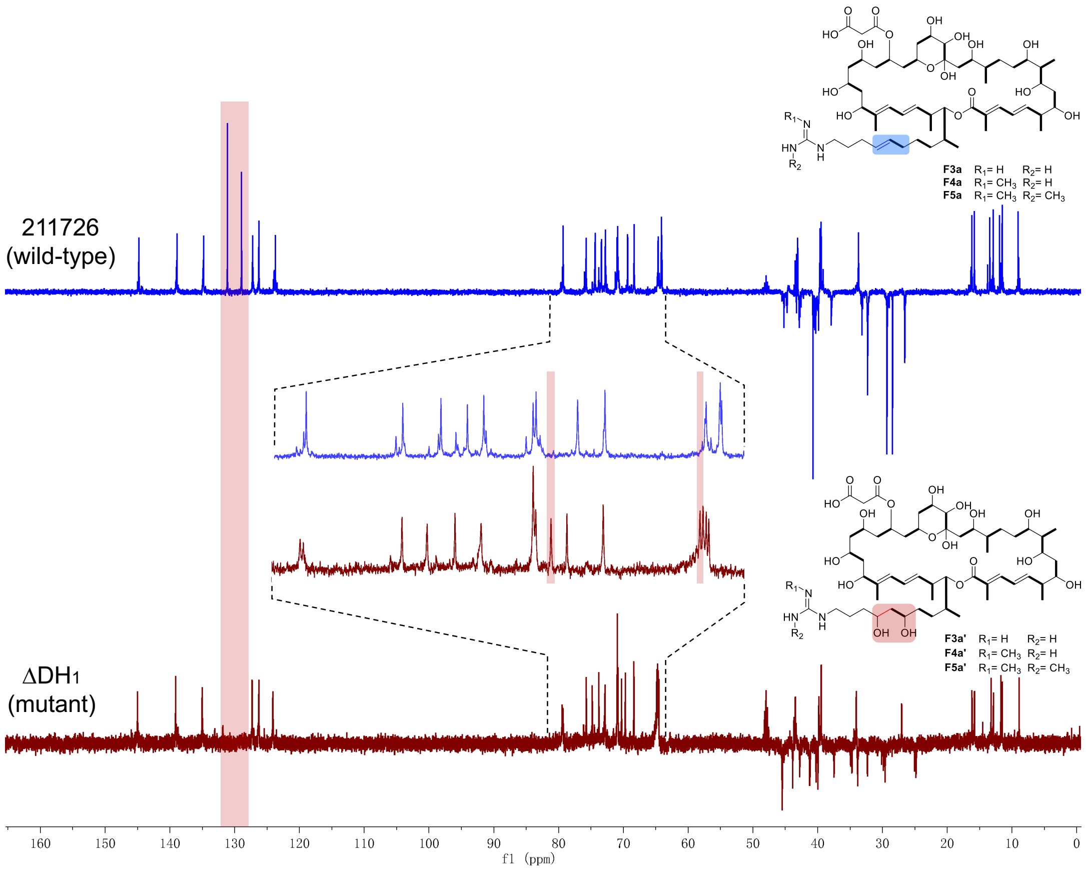

Figure S6. Comparison of DEPT spectra between AZL and derivatives produced by ΔDH_1 mutant.

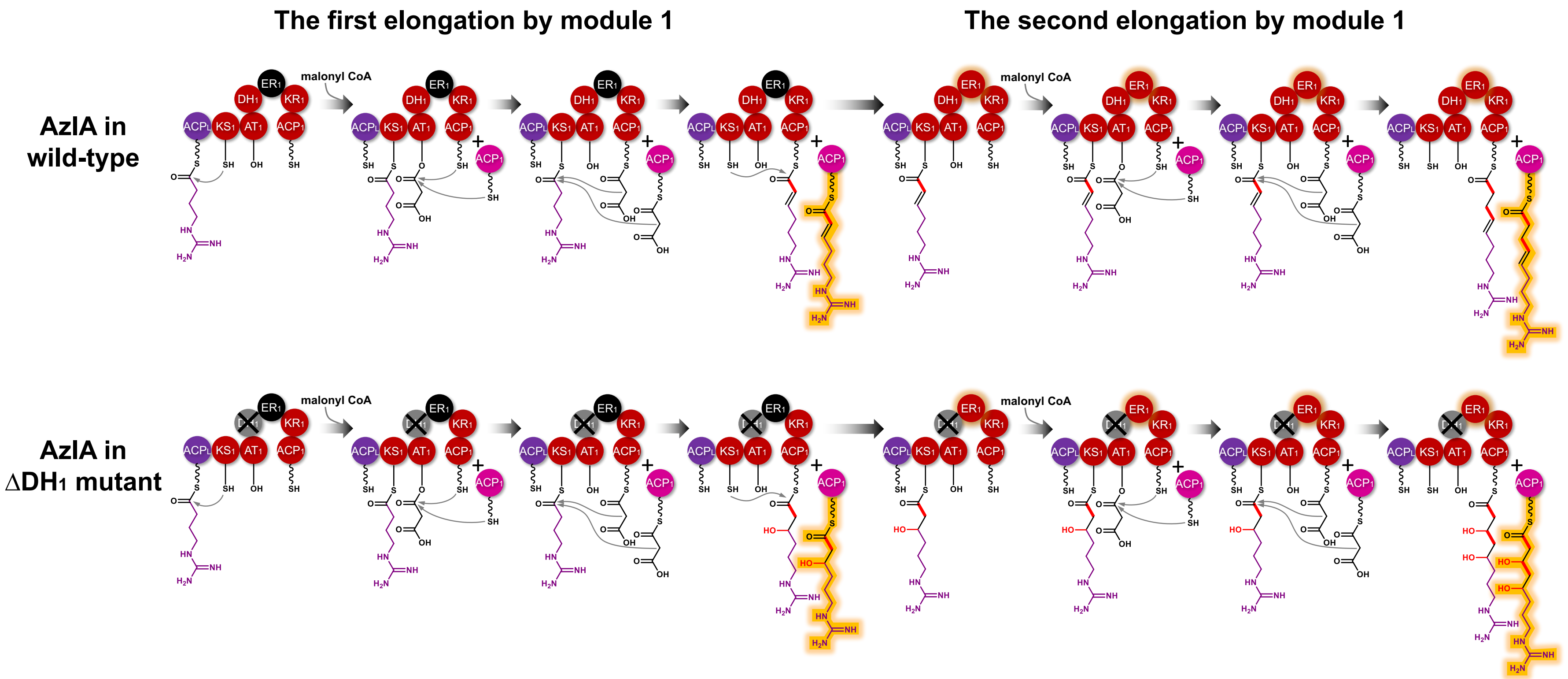

Figure S7. A detailed biochemical mechanism of module 1 processing in wild-type and ΔDH_1 mutant. In wild-type, 4-guanidinobutyryl-CoA, as the starter unit, is loaded onto loading domain (ACP_L) in AzlA by Azl5 (4-guanidinobutyryl-CoA:ACP acyltransferase), then transferred to the neighboring KS_1 domain of module 1. An extender unit, malonyl-CoA, is specifically selected by the AT_1 domain

followed by a transthioesterification to generate the acyl-S-ACP. Subsequently, the KS_1 domain catalyzes the Claisen-condensation reaction between starter and extender unit resulting in release of CO_2 and formation of a β-keto ester intermediate. Following this, the nascent polyketide chain is reduced stepwise to yield hydroxyl and enoyl group by KR_1 and DH_1 domain, respectively, without further reduction by ER_1 due to domain switch-off in some way. Notably, after the first elongation, the polyketide chain is retrotransferred to KS_1 for an iterative extension rather than normally to KS_2 of the next module. During the second elongation by the same module 1, all steps are the same as first one, except the enoylreduction by ER_1 is switched on to form a fully reduced ketide unit. In contrast, the subsequent reduction is halted after KR_1 reaction in the DH_1 inactivation mutant, leaving one or two hydroxyl groups in the polyketide chain. To better monitor the change of chain on the giant PKS by LC-ESI-HRMS, an additional recombinant *holo*-ACP_1 domain dissociated from module 1 was added in the in vitro assay, which allow to compete the growing polyketide chain with the integral ACP_1 domain of module 1. The captured intermediates by the dissociated ACP_1 is highlighted in yellow.

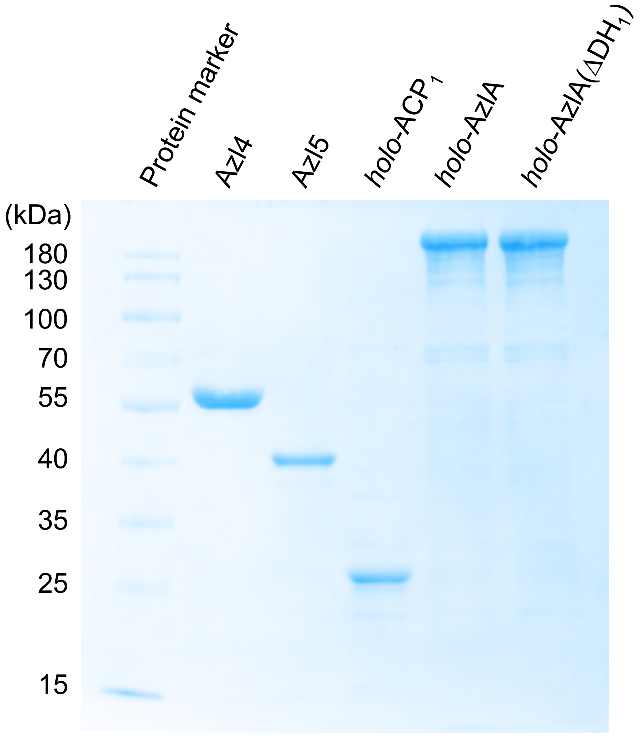

Figure S8. SDS-PAGE analysis of recombinant enzymes in this work. The theoretical molecular weights of Azl4, Azl5, *holo*-ACP$_1$, *holo*-AzlA and *holo*-AzlA(ΔDH$_1$) are 54023, 40856, 23660, 244455 and 244389 Da, respectively.

Table S1 *orfs* probably involved in AZL biosynthesis.

orf	**Size (bp)**	**Proposed function**
azl1	4749	Membrane protein
azl2	1176	Cytochrome P450
azl3	207	Ferredoxin
azlB	15576	PKS: module2 (KS-ATp-DH-KR-ACP); module 3 (KS-ATp-KR-ACP); module 4 (KS-ATa-DH-KR-ACP)
azlC	10104	PKS: module 5 (KS-ATp-DH-KR-ACP); module 6 (KS-ATa-KR-ACP)
azlD	14343	PKS: module 7 (KS-ATa-KR-ACP); module 8 (KS-ATa-KR-ACP); module 9 (KS-ATa-KR-ACP)
azlE	24795	PKS: module 10 (KS-ATa-KR-ACP); module 11 (KS-ATa-KR-ACP); module 12 (KS-ATa-DH-ACP); module 13 (KS-ATp-KR-ACP); module 14 (KS-ATa-DH-ER-KR-ACP)
azlF	9614	PKS: module 15 (KS-ATp-KR-ACP); module 16 (KS-ATa-KR-ACP)
azlG	10371	PKS: module 17 (KS-ATp-KR-ACP); module 18 (KS-ATa-DH-KR-ACP)
azlH	6339	PKS: module 19 (KS-ATp-DH-KR-ACP-TE)
azlA	6900	PKS: loading module (ACP); module 1 (KS-ATa-DH-ER-KR-ACP)
azl4	1437	4-guanidinobutanoate:CoA ligase
azl5	1035	4-guanidinobutyryl-CoA:ACP acyltransferase
azl6	609	TetR-family transcriptional regulator
azl7	885	Hydrolase
azl8	381	Hxl-family transcriptional regulator
azl9	408	Endoribonuclease L-PSP

azl10	789	Cellulose-binding family II
azl11	1281	Unknown protein
azl12	801	GntR-family transcriptional regulator
azl13	804	4-guanidinobutyramide hydrolase/Amidase[3]
azl14	1437	Amino acid permease
azl15	867	AraC-family transcriptional regulator

Table S2. Bacterial strains and plasmids used in this study.

Strains/Plasmids	Characteristics	Reference
E. coli		
DH10B	Host for general cloning	Invitrogen
ET12567/pUZ8002	Donor strain for conjugation between *E. coli* and *Streptomyces*	[4]
EPI300	Host for constructing the gene library	Epicentre
BL21 (DE3)	Host for protein expression	Invitrogen
BAP1	Host for protein expression	[5]
Streptomyces		
Streptomyces sp. 211726	Wild-type, azalomycin F (AZL) producing strain	[6,7]
∆azl	AZL biosynthetic gene cluster (*azl1* to *azl15*) deletion mutant, AZL non-producing	This work
∆azl4	*azl4* gene in-frame deletion mutant, AZL non-producing	This work
∆azl5	*azl5* gene in-frame deletion mutant, AZL non-producing	This work

ΔDH	Site-directed mutant of DH domain in *azlA*	This work
**Plasmid**		
pWHU2790	*azl* gene cluster deletion construct	This work
pWHU2791a	DH domain in module 1 inactivation construct	This work
pWHU2791b	DH domain in module 1 inactivation construct in which the active sites of DH domain was site-directed mutated	This work
pWHU106	Azl4 protein expression construct with N-terminal His-tag based on pET28a(+)	This work
pWHU107	Azl5 protein expression construct with N-terminal His-tag based on pET28a(+)	This work
pWHU86	AzlA protein expression construct with N-terminal His-tag based on pET28a(+)	This work
pWHU2793	AzlA protein expression construct with N-terminal His-tag based on pET28a(+), containing a single site-specific mutations (H1078A) in DH_1 domain	This work
pWHU2796	ACP_1 domain expression construct with N-terminal His-tag based on pET28a(+)	This work

Table S3. List of oligonucleotide primers used in this study.

Primer	Oligonucleotide sequences (5’ to 3’)	Restriction site
Δazl-L1	CAC**CATATG**CGACGACCCCGAGGAC	*Nde*I
Δazl-L2	ACC**GAATTC**CCCGAAGAGGTCTTCG	*Eco*RI
Δazl-R1	CTC**GAATTC**CTTCCTTGACGCTGAT	*Eco*RI
Δazl-R2	CTG**AAGCTT**TACGACGACATCACCG	*Hin*dIII
Δazl-CP1	ACAACACGTCGAGCAGGGAA	
Δazl-CP2	CGGTACGGACCTACGGATGG	
ΔDH1-L1	CCAGGGCGTGACGACCTGCG	
ΔDH1-L2	CACG**GCcgcGTCGGC**GAGCCACGGG	*Nco*I
ΔDH1-R1	**GCCGACgcgGC**CGTGGCCGGCGGAG	*Nco*I
ΔDH1-R2	CCGTAGCCGTAACCGACCTG	
ΔDH1-CP1	ACGGCGTCGGCGTGGACTGGGA	
ΔDH1-CP2	GGCCAGGCCCTCGTAGAAGC	
exAzl4-F	AAC**CATATG**ACCGCAAAGGTCTTTG	*Nde*I
exAzl4-R	TCG**GAATTC**TGTGGCTCGGTGTTCT	*Eco*RI
exAzl5-F	GAA**CATATG**ACGGACCCGCAGAAC	*Nde*I
exAzl5-R	AGT**GAATTC**CCATCGATCAGTCG	*Eco*RI
exAzlA-F	AGT**CATATG**AGCACCATGTTGAACG	*Nde*I
exAzlA-R	CTT**GAATTC**TCTTGGGGTGAGAGCC	*Eco*RI
exACP1-F	TCC**CATATG**AGCGGCCAGACCGAC	*Nde*I
exACP1-R	CTC**GAATTC**GGGGAGTCGAGATCCC	*Eco*RI

References

[1] Y. Sun, X. He, J. Liang, X. Zhou, Z. Deng, *Appl Microbiol Biotechnol* **2009**, *82*, 303–310.

[2] B. Gust, G. L. Challis, K. Fowler, T. Kieser, K. F. Chater, *Proc Natl Acad Sci U S A* **2003**, *100*, 1541–1546.

[3] Y. Ma, W. Xu, J. Zhang, S. Zhang, K. Hong, Z. Deng, Y. Sun, *Appl Microbiol Biotechnol* **2014**, DOI 10.1007/s00253-014-5762-z.

[4] D. J. MacNeil, J. L. Occi, K. M. Gewain, T. MacNeil, P. H. Gibbons, C. L. Ruby, S. J. Danis, *Gene* **1992**, *115*, 119–125.

[5] B. A. Pfeifer, S. J. Admiraal, H. Gramajo, D. E. Cane, C. Khosla, *Science* **2001**, *291*, 1790–1792.

[6] G. Yuan, H. Lin, C. Wang, K. Hong, Y. Liu, J. Li, *Magn Reson Chem* **2011**, *49*, 30–37.

[7] G. Yuan, K. Hong, H. Lin, Z. She, J. Li, *Marine Drugs* **2013**, *11*, 817–829.
